# Performance of Exergetic, Energetic and Techno-Economic Analyses on a Gas-Type Industrial Drying System of Black Tea

**DOI:** 10.3390/foods11203281

**Published:** 2022-10-20

**Authors:** Zhiheng Zeng, Bin Li, Chongyang Han, Weibin Wu, Tianci Chen, Chengju Dong, Changlun Gao, Zhaokai He, Fangren Zhang

**Affiliations:** 1 College of Engineering, South China Agricultural University, Guangzhou 510642, China; 2 School of Intelligent Manufacturing Engineering, Chongqing University of Arts and Sciences, Chongqing 404100, China

**Keywords:** exergetic, energetic, techno-economic, drying, tea, industrial

## Abstract

The purpose of this research work is to perform detailed exergetic, energetic and techno-economic analysis of the black tea drying process in the gas-type industrial dryer. Exergy–energy and techno-economic methodology was applied to investigate the heat loss, exergetic and energetic performance, exergy efficiency, improvement potential rate, sustainability index and techno-economic performance of a drying system. The results showed that the heat loss of exhaust air in the late drying process played a main contributing role in the heat loss and exergy loss of the whole drying system. Therefore, the exergy efficiency of the initial drying period and the redrying period varied from 38.08% to 65.09% and 24.76% to 26.97%, respectively. In addition, the improvement potential rate and sustainability index of the whole system varied from 6.93 kW to 12.94 kW and 1.33 to 2.86, respectively. The improvement potential obtained in the present work indicated that the drying operation is greatly in need of exergy performance improvement. Finally, the net present value and payback period obtained from techno-economic analysis were 179,442.03 USD and 5.3 years, the result is useful for investors or contractors to refer to and make investment decisions.

## 1. Introduction

Drying is a traditional method which is widely used to extend the preservation of agricultural or marine products such as grains, bananas, cassava and rough rice [1,2,3,4]. Traditionally, drying is a high-energy-consumption operation that removes water from material with high moisture content by externally supplying energy [5]. The original drying method is natural drying, and the current large-scale industrial production drying involves burning a large amount of fossil fuels, as found from the literature reporting that the energy consumed by the drying industry accounts for about 15% of the country’s total energy consumption, and the drying cost in particular is close to 60–70% of the total cost [6,7]. Considering the global energy shortage and sustainable development strategies, it is necessary to reveal the overall energy matching structure and the transfer mechanism of the drying system, and clarify the evaluation indicators, such as energy efficiency and sustainability of the drying system. Therefore, it is of great significance to improve the overall efficiency of the drying system and develop the drying theory for the energy conservation and sustainability of the drying industry.

The drying process of material is a dewatering process based on the supply of heat. From the perspective of thermodynamics, the drying of materials is due to the heat transfer which increases the activation energy of water molecules to form a temperature field and a humidity field, which promotes the evaporation and dehydration of the moisture inside the material [8,9]. As mentioned above, the drying processes is a highly energy-intensive operation and the kinetic process of mass transfer and heat transfer. Application of the first and second laws of thermodynamics to reveal the energy structure and transfer mechanism of the agricultural product drying system or industrial production drying system is a method commonly used by researchers in recent decades [10,11,12]. For example, Silva et al. designed and tested a solar dryer of corn grains and evaluated the performance of energy and exergy of the dryer based on the first and second laws of the thermodynamics, identified that the average thermal efficiency was 21% and the exergy efficiency ranged between 10–66% [13]. Not only that, there are many such research studies on the evaluation and analysis of agricultural product drying system by the exergy analysis method, such as bananas [14] and medicinal plants [15]. The drying systems mentioned above are laboratory-scale drying systems, and there are also a few studies in the literature which reported industrial-scale drying systems [16,17,18]. Sarker et al. and Li et al. employ the method of energy–exergy to estimate the energetic and exergetic performance of a novel industrial multi-field synergistic dryer, and carried out the influence of the crackle ratio, generation potential, and generation rate for the quality of the dried paddy [16]. Moreover, energy is a quantitative indicator which is defined as the amount of external work applied by a system. Different to energy, exergy is a qualitative indicator which is defined as the maximum useful work supplied by a system externally [19,20,21]. The method of energy–exergy analysis not only enables the energy efficiency of the drying system to be evaluated in terms of energy quantity, but also the exergy of different components of the system can be evaluated in terms of energy quality to help make informed design decisions [22]. Therefore, obtaining energy (exergy) efficiency based on the energy–exergy analysis method can not only evaluate the sustainability of the drying system, but also facilitate the guidance of the design of efficient dryers [23].

For a drying system, the evaluation of energy and efficiency is essential. Similarly, the overall economics of drying production are also a considerable indicator that should be fully considered for producers or farmers. The economic estimation of the drying system consists of the investment cost and the production cost of the drying system. However, the production cost may be controversial, as the comprehensive availability of local raw materials, the cost of local manufacturing, and the cost of power sources deviate between places [24]. Currently, economics evaluation is the primary and most common method for drying systems, while the techno-economic method is among the most common and popular economic analysis methods for studying thermodynamic systems, such as the traditional energy system [25,26] and emerging energy system [27,28]. The main advantages of the techno-economic method are that the cost flow and the energy flow of the system can be combined to identify the energy–exergy destruction rate of each component, indicate the improvement direction, and obtain the unit cost of the final product of the system [29]. The evaluation factor which is carried out based on the thermodynamic–economic considerations mentioned above cannot fully meet the requirements of economic evaluation of a drying system which undertakes the large task of high-production drying quantities every year. For investors or contractors, the economic rationality of the equipment and operating costs required for the drying system should receive more attention. Therefore, a few relevant studies in the literature [10,30,31,32] have reported a method which applied a techno-economics indicator, such as capital cost (*C_c_*), payback period (*PP*), and net present value (*NPV*), etc., to evaluate the performance of the techno-economics of a drying system. For instance, Yahya et al. applied the techno-economic analysis method to obtain the payback period and net present value of the solar heat pump fluidized bed dryer with the value of 1.6 years and 8563.82 dollars, respectively [33]. Nadiya et al. have reported detailed economic analysis through the annualized cost, life cycle saving and payback period method [34]. Moreover, economic estimation used the macroeconomics indicator mentioned above to provide a simple method for investors or contractors to deal with simple economic issues and make the correct choice for the selection and use of drying systems [35,36]. Therefore, investors or contractors can determine the overall rate of return and payback period of the drying system and evaluate various technical options to meet the system based on the overall capital cost, the whole revenue and profit of the drying system obtained by economic analysis.

To summarize, in the last few decades, evaluation of the exergy–energy and techno-economic indicators was widely applied to evaluating the performance of the drying system. In the present work, we pay attention to a gas-type industrial drying system of black tea, which is currently the most widely used tea dryer [37], whereas relevant research studies have carried out research on the thin-layer drying model of tea, the prediction of drying time, and the heat and mass transfer model for the tea dryer [38,39,40,41], knowledge of performance analysis on exergy–energy and the techno-economics of the gas-type industrial drying system of black tea seems to be scarce in the existing scientific research literature. Therefore, the objective of the present study is not only concerning performance analysis of exergetic and energetic factors for a gas-type industrial drying system of black tea based on the first and second law of thermodynamics, which to reveal the energy loss in the drying system and to guide to design more efficient thermal systems, but also evaluating indicators such as net present value (*NPV*) and payback period (*PP*) to assess the economic performance of the drying system from the techno-economic perspective. This will generate insight into the drying system performance, benefitting the whole revenue stream by increasing energy utilization and commercialization of the drying system for an investor or contractor.

## 2. Materials and Methods

### 2.1. Materials

**Figure 1:**
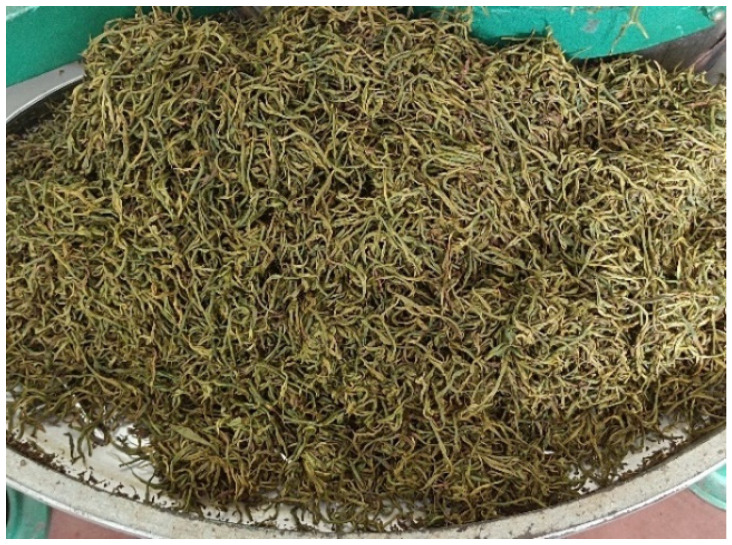
The freshly tea leaves for the experiment.

The experimental material shown in Figure 1 was freshly tea leaves (variety: Yinghong NO. 9) with an average initial moisture content 58.33% (w.b.), harvest from a local farm at Yangshan County, Guangdong Province.

### 2.2. Operation Principle of Experimental

**Figure 2:**
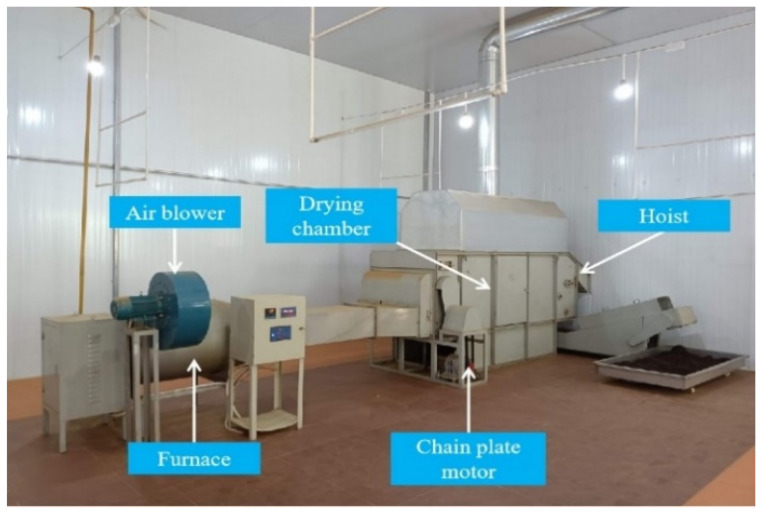
Photograph of the gas-type industrial drying system of black tea.

A gas-type industrial drying system of black tea was installed in a tea enterprise in Yangshan County, Guangdong Province. The photograph of a gas-type industrial drying system of black tea was shown in Figure 2, which consisted of five principal components, including an air blower, furnace, drying chamber, hoist and chain plate motor.

**Figure 3:**
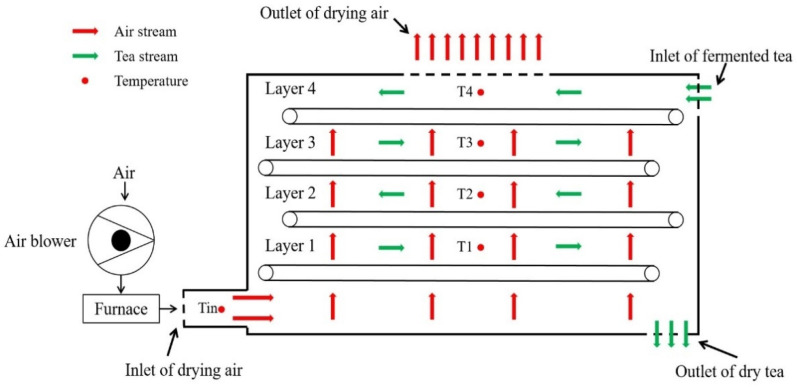
Schematic sketch of the gas-type industrial drying system of black tea.

Experiments were carried out in a tea enterprise in Yangshan County, Guangdong Province. The schematic diagram of a gas-type industrial drying system of black tea is shown in Figure 3. Tea farmers freshly harvested tea in tea garden, and turned it into approximately 180 kg of fermented tea leaves, which were placed into the drying chamber for the drying process. The working principle of the gas-type industrial drying system of black tea is that the air blower sends the air into the furnace for heating and then sends the heated air into the drying chamber from the bottom of the left side of the drying chamber to dry the fermented tea leaves which enter the conveyor chain network from the top feed port. The drying leaf reciprocates with the chain mesh layer to send the material to the discharge port. The hot-air-dried the fermented tea leaves are discharged from the exhaust pipe at the top of the drying chamber. Generally, the operation of drying was divided into two periods, the initial drying period and the redrying period. The detailed operation process is shown in Table 1.

The temperature of the hot air at the inlet of the drying chamber is measured by a temperature sensor inserted into the hot air inlet and displayed in the control cabinet of the combustion chamber. Air temperature in the drying chamber was measured by using a PT100 thermal resistance. During the overall drying operation, the temperatures of the four layers of the drying chamber (T_i_, i = 1, 2, 3 and 4, acquiescence T_4_ = T_outlet_ especially) were measured by thermal resistance sensors inserted into the corresponding components. The air velocity and pressure were measured by using a wind speed and air volume measuring instrument. The details of the relevant measuring instruments are listed in Table 2.

Measurement equipment error, data collection operation error and data accuracy will all have a certain impact on the accuracy of the overall work [42]. Therefore, the ascertainment of uncertainty analysis is necessary, and the calculation formula is as follows:

(1) $$W={\left[{\left(\frac{\partial y}{\partial x_{1}}w_{1}\right)}^{2}+{\left(\frac{\partial y}{\partial x_{2}}w_{2}\right)}^{2}+\ldots +{\left(\frac{\partial y}{\partial x_{n}}w_{n}\right)}^{2}\right]}^{1/2}$$

where *W* is total uncertainty in result measurement, *w*_1_, *w*_2_, … *w_n_* are uncertainties in independent variables and *x*_1_, *x*_2_, … *x_n_* are independent variables.

### 2.3. Energy–Exergy Analysis

In this work, the mass and energy balance equation were applied to analyze the performance of the energy and exergy in a gas-type industrial drying system of black tea which were defaulted to be a steady state and steady flow drying system, the relevant calculation formula is as follows [43]:

(2) $$\sum m_{in}h_{in}=\sum m_{out}h_{out}\;$$

The energy conversation of the whole system can be expressed as the following calculation [40]:

(3) $$\sum Q+\sum m_{in}h_{in}=\sum W+\sum P+\sum m_{out}h_{out}$$

In the present work, the total heat (*Q_gas_*) supplied by natural gas to the drying system, the heat (*Q_a_*) used for heating the natural air, the useful energy (*Ex_gas_*) to heat the air, the power for the air blower (*P_blower_*), the chain plate motor (*P_motor_*), the hoist (*P_hoist_*), the specific energy consumption (*SEC*), the specific thermal energy consumption (*STEC*), the exergy efficiency for the drying chamber (*η_ex_*), the improvement potential (*IP*) and the sustainability index (*SI*) of the drying system can be calculated using Formulas (4)–(19) tabulated in Table 3 [44,45,46].

### 2.4. Economic Analysis

In the present work, in addition to the performance of exergetic and energetic indicators, the economics of the black tea drying system, consisting of the whole capital cost (*C_c_*) and the product cost (*C_p_*), must be considered. The investment cost of the dryer in the system mainly includes the air blower, furnace, drying chamber, chain plate motor, hoist, cost for manual installation and infrastructure cost. Additionally, the production cost consists of the electricity, gas, labor, maintenance and depreciation. In addition, the profit (*PR*), return of capital (*ROR*), payback period (*PP*), net present value (*NPV*) and present value (*S*) of the drying system can be calculated using Equations (20)–(25) shown in Table 4 [29,47].

Where in the table, *C_el_*, *C_gas_*, *C_la_*, *C_m_* and *C_dp_* are the cost of electricity, gas, labor, maintenance and depreciation; *P_n_* is the discounted present value (*S*) of the investment to be invested in the next *n* years.

## 3. Results and Discussion

### 3.1. Energy Matching Structure of the Drying System

The uncertainty analysis of the parameters in the drying process are shown in Table 5. Compared with the previous studies [48,49], uncertainties regarding the experimental data were within a reasonable range, indicated that the dependability of the data used for calculating the indicators adopted in the present work.

**Figure 4:**
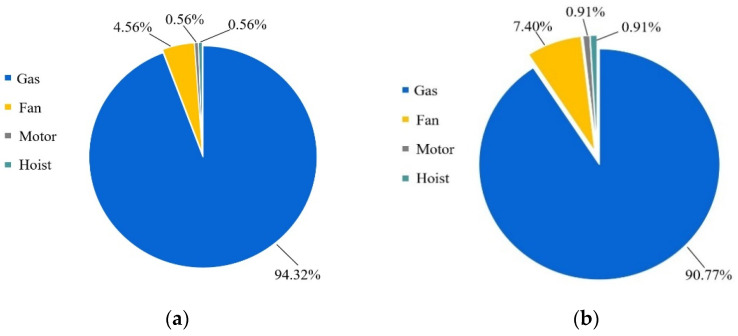
Total energy consumption ratios of the whole system: (**a**) energy consumption ratios in initial drying period; (**b**) energy consumption ratios in redrying period.

In the present work, drying operation consumption mainly include the energy of electricity and natural gas. The heat required to dry the material of the drying system supplied by the natural gas, the whole drying machine is provided by electricity. Detailed parameters of the related equipment applied in the present study are listed in Table 6. Furthermore, Figure 4 describes the proportion of the initial drying period, the redrying period energy, as well as the whole energy consumption of the drying system and energy consumption, including gas, induced fan and hoist. In the initial drying period, energy consumption by gas, fan, motor and hoist account for 94.32%, 4.56%, 0.56% and 0.56% in the initial drying system, in the redrying system account for 90.77%, 7.40%, 0.91% and 0.91%, respectively.

### 3.2. Heat Loss Characteristics of the Drying System

Heat loss is an important indicator to give reasons for the high energy consumption of drying systems. To clear energy consumption and energy efficiency of the system, the heat loss characteristics of the drying system were investigated. In the present study, the heat loss of the drying system mainly considers the heat loss caused by the exhaust air outlet (*Q_loss,air_*) and the heat loss of wall heat transfer (*Q_loss,wall_*), the relevant calculation results shown in Table 7. As can be seen from Table 7, whatever in the initial drying period or redrying period, the heat loss of the exhaust air outlet played a main contributing role in the heat loss of the drying system. The value of the variation of the heat loss of exhaust air is 14.89–52.42 kW in the initial drying period and 49.55–51.27 kW in the redrying period; the value of the heat loss of wall heat transfer is 0.946–1.421 kW in the initial drying period and 1.227–1.240 kW in the redrying period. In addition, the variation interval of the whole heat loss is 15.84–53.84 kW in the initial drying period and 50.78–52.51 kW in the redrying period. The change trend of heat loss during the drying period of the whole drying system is that the heat loss of the exhaust gas and wall heat transfer in the initial drying period increases with the increase in drying time, and then tends to be stable until the redrying period and the end of drying. In the beginning of the drying process, the evaporation of the water of the fresh tea required the consumption of a lot of heat, and instability of the drying chamber temperature leads to the lower heat loss. As the drying process progresses, the variation curve of heat loss tends to be constant, which may be the result of the drying chamber temperature tending towards stability and the outlet temperature being higher than the temperature of the tea. The situation of the heat loss of the whole drying system tending to be steady in the latter period of the drying has been reported in relevant literature [50,51,52]. Thus, efforts should be made to recover and reuse the heat of the exhaust gas to improve the energy efficiency of the whole drying system.

### 3.3. Analysis the Exergy Flow of the Drying System

**Figure 5:**
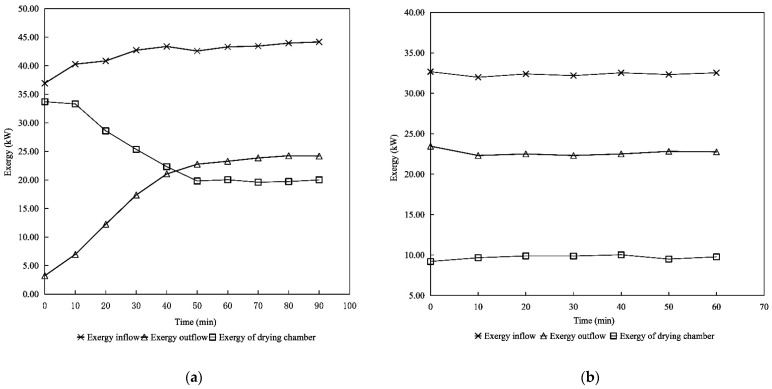
Variation in the exergy inflow, exergy outflow and exergy of the chamber vs. drying time: (**a**) variation of exergy in the initial drying period; (**b**) variation of exergy in the redrying period.

The corresponding change trend curve of exergy flow is shown in Figure 5. Figure 5 describes the trend of the curve of the exergy inflow and exergy outflow increasing with the increase in time, and then tending to stability until to end of the drying. In the initial drying period, the value of the exergy inflow, exergy outflow and exergy of the drying chamber varied between 36.94–44.18 kW, 3.23–24.24 kW and 19.72–33.70 kW, respectively; and in the redrying period varied between 31.99–32.67 kW, 22.50–22.76 kW and 9.21–10.03 kW, respectively. Comparing with Table 7, we can see that the trend of the exergy flow is similar to the trend of the heat loss flow. In the beginning of the drying process, the temperature of the tea entering the drying chamber is lower, which results in huge amounts of exergy consumed to increase the temperature of the tea and remove moisture. Furthermore, the increase in the average temperature of the whole drying chamber results in the increase in the exergy outflow. A similar investigation has been made by Beigi for rice in a deep-bed convective dryer [53]. In general, exergy loss of the outflow played a contributing role in the exergy loss of the whole drying system, hence, a few studies in the literature have indicated that the exergy loss from the exergy outflow to the surroundings is one of the thermodynamic inefficiencies of drying systems [54,55]. Therefore, the focus of effort is not only on the heat recovery of exhaust air, but also on the exergy loss of the exergy outflow to further optimize the structure of the drying system.

### 3.4. Exergetic Performance of the Drying System

In the present study, evaluation of the indicators, including exergy efficiency (*η_ex_*), improvement potential (*IP*), sustainability index (*SI*), specific energy consumption (*SEC*) and specific thermal energy consumption (*STEC*), was carried out to investigate the exergetic performance of the drying system. The variations in the evaluation indicators mentioned above are shown in Table 8 during the whole drying period.

As shown in Table 8, *η_ex_* and *SI* decrease with the increase in the drying time; conversely, *IP* increased with the increase in the drying time. In the present work, exergy efficiency values varied from 37.85% to 65.09% in the initial drying period and 24.76% to 26.97% in the redrying period. According to the previous analysis of the change trend of heat loss and exergy flow, the exergy efficiency increased with the decrease in the heat loss and exergy outflow. It can be clearly seen from Table 8 that the improvement potential (*IP*) values change from 11.77 kW to 12.94 kW in the initial drying period, and from 6.93–7.33 kW in the redrying period. Additionally, corresponding to the initial and redrying period, the value of the sustainability index of the present study varied from 1.61 to 2.96 and from 1.33 to 1.37. Previous studies have indicated that the higher exergy efficiency resulted the higher sustainability index and the lower effect on the environment [56]. Accordingly, efforts should be made to ameliorate the exergy efficiency of the present drying system to reduce the effective on the environment. Furthermore, as can be clearly seen from Table 8, the value of *SEC* and *STEC* varied from 18.09 kJ/g to 21.31 kJ/g and 16.43 kJ/g to 21.21 kJ/g in the initial drying period; but in the redrying period, the value of *SEC* and *STEC* varied from 127.13 kJ/g to 129.58 kJ/g and 113.82 kJ/g to 116.27 kJ/g, respectively. The phenomenon that *SEC* and *STEC* increases rapidly in the redrying period due to the existence of the binding energy, and a similar conclusion was found by Xiong et al. [57] and Yildirim et al. [58].

### 3.5. Techno-Economic Analysis of the Drying System

In this section, the techno-economic analysis mainly includes the capital cost of the drying system, the net present value, the net cash flow and the payback period; the following Table 9, Table 10, Table 11, Table 12 and Table 13 record the economic analysis results of the whole drying system of the present work. The discount factor, maintenance costs, depreciation rate and expected life of the dryer were performed with an assumed value of 10%, 2%, 9.5% and 10 years, respectively. As can been seen from Table 9, the cost of infrastructure and tea garden construction and management constitute the main component of the whole capital cost. Based on the capital costs mentioned above and the parameters shown in Table 10, the product costs include the cost of fresh tea (30,731.04 USD/Year), labor (21,865.89 USD/Year), electricity (139.97 USD/year), natural gas (8388.06 USD/year), maintenance (3147.01 USD/year) and depreciation (14,948.32 USD/year). Table 11 has tabulated annual operation cost, annual revenue and the net cash flow were 64,271.97 USD/year, 94,413.60 USD/year and 30,141.63 USD/year, respectively. As can be shown in Table 12 and Table 13, the economic analysis of the drying system in the present work calculated and demonstrated that the net present value and the payback period had values of 179,442.03 USD and 5.3 Years, respectively.

## 4. Conclusions

In the present work, black tea was dried through the use of a gas-type industrial dryer. In the aspect of energy efficiency evaluation of the drying system, the exergetic and energetic performance of the dryer were investigated; in the aspect of economic analysis of the whole system, the net present value and payback period of the system were considered in the investigation. The main conclusion to be drawn are as follows:

1. In approximately the first 50 min of the whole drying process, both the heat loss and exergy outflow increase with the increase in drying time, and then become stable as a result of the influence of the binding energy between the dry matter and water molecules until to end of drying process.
2. The heat loss of the exhaust air makes an important contribution to the heat loss and exergy loss of the whole drying system, especially in the redrying period. Therefore, it is recommended that a device be designed to recover heat from the exhaust air so as to improve the energy efficiency and reduce the exergy loss rate of the whole drying system.
3. In the drying process, the improvement potential rate varied from 6.93 kW to 12.94 kW and the value of the sustainability index ranged from 1.33 to 2.86 of the drying system, indicating that the exergy efficiency should be improved so as to improve the environmental sustainability.
4. According to the techno-economic method’s calculations, the net present value and payback period are 179,442.03 USD and 5.3 years, respectively.

Finally, further studies are advised to investigate the influence of factors such as ventilation temperature and speed on the drying efficiency, optimize the drying process parameters, and further improve the sustainability and efficiency of the drying system.

## Figures and Tables

**Table 1:** Detailing operation process in the present work.

Materials	Drying Period (Air Temperature)	Duration (min)	Mian Function	Product
Fresh tea leaves (Approximately 180 kg)	Initial drying (120 °C)	90 min	Initial drying tea	Dry tea (Approximately 45 kg)
	Redrying (100 °C)	60 min	Redrying tea	

**Table 2:** Details of the experimental instruments.

Instrument	Type	Measurement	Instrument
Thermal resistance	PT100	−200–450 °C	±0.1 °C
Temperature and humidity sensors	AM2301	0–100%/−40–80 °C	±3%/±0.5 °C
Electronic scale	ABJ 320-4NM	0–380 g	±0.01 g
Constant-temperature drying box	DGG-9070A	105 °C	±0.1 °C
Data acquisition system	Self-developed	-	-
Intelligent anemometer	DP2000	0~100 m/s, 0~ + ∞ m^3^/h, 0~ ± 10 KPa	±0.1 m/s, ±0.5 m^3^/h, ±0.5 KPa

**Table 3:** Energy–exergy analysis of black tea drying system.

Energy–Exergy Analysis	Equation	Eq. No.
Total heat applied from gas	$Q_{gas}=V_{gas}\times q_{gas}$	(4)
Energy for heating air	$Q_{a}=m_{g,a}c_{p,a}\left(T_{1}-T_{0}\right)$	(5)
Heat loss of exhaust air outlet	$Q_{loss,air}=m_{g,a}c_{p,a}\left(T_{4}-T_{0}\right)$	(6)
Heat loss of the wall heat transfer	$Q_{loss,wall}=\frac{1.3A_{0}}{\left(\delta /\gamma \right)}\overset{4}{\underset{i=1}{\sum }}\left(T_{wall,i}-T_{0}\right)$	(7)
Exergy for heating air	$Ex_{gas}=m_{g,a}c_{p,a}\left[\left(T_{1}-T_{0}\right)-T_{0}ln\left(\frac{T_{1}}{T_{0}}\right)\right]$	(8)
Exergy of air blower	$Ex_{blower}=W_{blower}=P_{blower}t$	(9)
Exergy of chain plate motor	$Ex_{motor}=W_{motor}=P_{motor}t$	(10)
Exergy of hoist	$Ex_{hoist}=W_{hoist}=P_{hoist}t$	(11)
Total exergy entering the drying system	$Ex_{dc,in}=Ex_{gas}+Ex_{blower}+Ex_{motor}+Ex_{hoist}$	(12)
Total exergy outlet the drying system		(13)
The specific energy consumption	$SEC=\frac{Ex_{gas}+Ex_{blower}+Ex_{motor}+Ex_{hoist}}{DR}$	(14)
The specific thermal energy consumption	$STEC=\frac{Ex_{gas}}{DR}$	(15)
Exergy of drying chamber	$Ex_{dc}=Ex_{dc,in}-Ex_{dc,out}$	(16)
Exergy efficiency for drying chamber	$\eta_{ex}=\frac{Ex_{dc}}{Ex_{dc,in}}$	(17)
The improvement potential	$IP=\left(1-\eta_{ex}\right)Ex_{dc}$	(18)
The sustainability index	$SI=\frac{1}{\left(1-\eta_{ex}\right)}$	(19)

**Table 4:** Economic analysis of black tea drying system.

Economic Index	Equation	Eq. No.
Production cost	$C_{P}=C_{el}+C_{gas}+C_{la}+C_{m}+C_{dP}$	(20)
Profit	$PR=TS-C_{C}-C_{P}$	(21)
Return of capital	$ROR=\frac{PR}{C_{C}}$	(22)
Payback period	$PP=\frac{C_{C}}{PR}$	(23)
Net present value	$NPV=\overset{N}{\underset{n-1}{\sum }}P_{n}{\left(1+i\right)}^{n}-C_{C}$	(24)
	$P_{n}=S{\left(1+i\right)}^{-n}$	(25)

**Table 5:** Detail parameters of the related equipment applied in this work.

Parameters	Unit	Uncertainty Value
Moisture content	g_water_/g_wet mateer_	±0.012
Air temperature	°C	±0.299
Mass of the product	kg	±0.113
Specific energy consumption	kJ/g	±4.436%
specific thermal energy consumption	kJ/g	±4.391%
Time measurement	min	±0.124

**Table 6:** Detailed parameters of the related equipment applied in this work.

Equipment	Power
Induced fan	3 kw
Chain plate motor	0.37 kw
Hoist	0.37 kw

**Table 7:** Variation of the heat loss in the different drying period.

Period	Initial Drying Period										Redrying Period						
Time (min)	0	10	20	30	40	50	60	70	80	90	100	110	120	130	140	150	160
Heat loss of exhaust air (kW)	14.89	23.49	33.42	41.91	47.64	50.22	50.98	51.84	52.42	52.32	51.27	49.55	49.84	49.55	49.84	50.32	50.22
Heat loss of wall heat transfer (kW)	0.946	1.127	1.214	1.315	1.375	1.378	1.398	1.415	1.420	1.421	1.240	1.227	1.231	1.227	1.230	1.230	1.229
Percentage of total heat loss to total heat (%)	14.38	22.34	31.44	39.24	44.49	46.84	47.55	48.34	48.87	48.78	57.20	55.31	55.63	55.31	55.63	56.15	56.04

**Table 8:** Variations in the evaluation indicators of the drying system.

Drying Process	Drying Time (min)	Drying System				
		*η_ex_*	*IP* (kW)	*SI* (-)	*SEC* (kJ/g)	*STEC* (kJ/g)
Initial drying period	0	65.09%	11.77	2.86	18.09	16.43
	10	64.34%	11.88	2.80	19.58	17.91
	20	55.23%	12.80	2.23	19.83	18.17
	30	48.97%	12.94	1.96	20.67	19.00
	40	43.12%	12.70	1.76	20.96	19.29
	50	38.28%	12.23	1.62	20.60	18.94
	60	38.69%	12.28	1.63	20.92	19.26
	70	37.85%	12.18	1.61	20.99	19.32
	80	38.08%	12.21	1.62	21.21	19.55
	90	38.63%	12.28	1.63	21.31	19.65
Redrying period	100	24.76%	6.93	1.33	129.58	116.27
	110	26.00%	7.16	1.35	127.13	113.82
	120	26.60%	7.26	1.36	128.60	115.29
	130	26.56%	7.26	1.36	127.87	114.56
	140	26.97%	7.33	1.37	129.09	115.78
	150	25.55%	7.08	1.34	128.36	115.05
	160	26.28%	7.21	1.36	129.09	115.78

**Table 9:** The capital costs of the drying system.

Item Cost	USD
Furnace	1025.94
Blower	180.47
Drying chamber	6414.00
Hoist	1676.38
Chain plate motor	94.75
Transportation and installation costs	728.86
Infrastructure cost	72,886.30
Tea garden construction and management costs	74,344.02
Whole capital cost	157,350.72
Currency exchange rate: 6.86 CNY = 1 USD	

**Table 10:** Parameters used in the calculation of the net present value (*NPV*) and payback period (*PP*) of the drying system.

Parameter	Unit	Value
Initial weight of tea	kg	180
Final weight of tea	kg	45
Initial moisture content of tea (wet basis)	%	58.33
Finial moisture content of tea (wet basis)	%	4.63
Drying time	min	162
Price of the fresh tea	USD/kg	0.67
Factory price of dried tea	USD/kg	14.57
Price of natural gas	USD/kg	3.12
Price of electricity	USD/kWh	0.10
Tea harvest and management costs	USD/day	213.41
Discount factor	%	10
Maintenance costs	%	2
Depreciation	%	9.5
Expected life of the dryer	Year	10
Net present value (*NPV*)	USD	179,442.03
Payback Period (*PP*)	Years	5.3
Currency exchange rate: 6.86 CNY = 1 USD		

**Table 11:** Cash flow statement of the drying system.

Years	0	1	2	3	4	5	6	7	8	9	10
Capital costs (USD)	157,350.72										
Production costs (USD)											
Fresh tea		21,865.89									
Labor		30,731.04									
Electrical		139.97									
Natural gas		8388.06									
Maintenance		3147.01									
Depreciation		14,240.24									
Total production costs (USD)		64,271.97									
Revenue (USD)		94,413.60									
Net cash flow (USD)		30,141.63									
Currency exchange rate: 6.86 CNY = 1 USD											

**Table 12:** *NPV* of the drying system (*NPV* = 179,442.03 USD).

Years	Capital Costs (CC)	Net Cash Flow	Discount Factor	Present Value
	(USD)	(USD)	(*i* = 10%)	(USD)
0	157,350.72		1	
1		30,141.63	0.9091	27,401.76
2		30,141.63	0.8264	24,909.04
3		30,141.63	0.7513	22,645.41
4		30,141.63	0.6830	20,586.73
5		30,141.63	0.6209	18,714.94
6		30,141.63	0.5645	17,014.95
7		30,141.63	0.5132	15,468.68
8		30,141.63	0.4665	14,061.07
9		30,141.63	0.4241	12,783.07
10		30,141.63	0.3855	11,619.60
				185,205.25
The final depreciation value		14,948.32		
The final present value		5763.22		
Currency exchange rate: 6.86 CNY = 1 USD				

**Table 13:** Payback Period of drying system (*PP* = 5.3 years).

Years	Capital Costs (CC)	Annual Benefit	Benefit Cumulative
	(USD)	(USD)	(USD)
0	157,350.72	0	0
1		30,141.63	30,141.63
2		30,141.63	60,283.26
3		30,141.63	90,424.89
4		30,141.63	120,566.52
5		30,141.63	150,708.15
6		30,141.63	180,849.78
Currency exchange rate: 6.86 CNY = 1 USD			

## Data Availability

Not applicable.

## Nomenclature

*m*	Mass flow rate (kg s^−1^)
*Ex*	Exergy rate (kW)
*h*	Specific enthalpy (kJ kg^−1^)
*Q_gas_*	Heat of gas (J)
*V_gas_*	Unit volume of natural gas (m^3^)
*Q_a_*	Heat of heating air (J)
*m_g_*	Mass flow of air (kg s^−1^)
*c_p_*	Specific heat of air (J kg^−1^ °C^−1^)
*T*_1_	Temperature of inlet (°C)
*T*_4_	Temperature of outlet (°C)
*T*_0_	Temperature of ambient (°C)
*T_wall_*	Temperature of wall (°C)
*Q_loss,air_*	Heat loss of exhaust air (kW)
*Q_loss,wall_*	Heat loss of the wall heat transfer (kW)
*A*_0_	Heat transfer area of the drying chamber (m^2^)
*Ex_gas_*	Exergy of gas (kW)
*Ex_blower_*	Exergy of blower (kW)
*Ex_motor_*	Exergy of chain plate motor (kW)
*Ex_hoist_*	Exergy of hoist (kW)
*Ex_dc,in_*	Total exergy entering the drying system (kW)
*Ex_dc,out_*	Total exergy outlet the drying system (kW)
*Ex_dc_*	Exergy of drying chamber (kW)
*W_blower_*	Work rate of blower (kW)
*W_motor_*	Work rate of chain plate motor (kW)
*W_hoist_*	Work rate of hoist (kW)
*P_blower_*	Power rate of blower (kW)
*P_motor_*	Power rate of chain plate motor (kW)
*P_hoist_*	Power rate of hoist (kW)
Abbreviations	
*DR*	Drying rate (g_water_/g_wet matter*h_)
*SEC*	Specific Energy Consumption (kJ/g)
*STEC*	Specific Thermal Energy Consumption (kJ/g)
*SI*	Sustainability index
*IP*	Improvement potential rate (kW)
*NPV*	Net present value
*PP*	Payback period
Subscripts	
*a*	Air
*in*	Inlet
*out*	Outlet
Greek symbols	
*η_ex_*	Exergy efficiency (%)
*δ*	Thickness (m)
*γ*	Thermal conductivity (W m^−1^ °K^−1^)
